# Association of Area Deprivation With Primary Hypertension Diagnosis Among Youth Medicaid Recipients in Delaware

**DOI:** 10.1001/jamanetworkopen.2023.3012

**Published:** 2023-03-15

**Authors:** Carissa M. Baker-Smith, Wei Yang, Mary J. McDuffie, Erin P. Nescott, Bethany J. Wolf, Cathy H. Wu, Zugui Zhang, Robert E. Akins

**Affiliations:** 1 Cardiovascular Research and Innovation Program, Nemours Cardiac Center, Nemours Children’s Health, Wilmington, Delaware; 2 Department of Biostatistics, Epidemiology and Informatics, University of Pennsylvania Perelman School of Medicine, Philadelphia; 3 Center for Community Research and Service, University of Delaware Biden School of Public Policy and Administration, University of Delaware, Newark; 4 Medical University of South Carolina, Charleston; 5 Data Science Institute, University of Delaware, Newark; 6 Institute for Research in Equity and Community Health, Christiana Care Health Services, Inc, Newark, Delaware; 7 Center for Pediatric Clinical Research and Development, Nemours Children’s Health, Wilmington, Delaware

## Abstract

**Importance:**

The association between degree of neighborhood deprivation and primary hypertension diagnosis in youth remains understudied.

**Objective:**

To assess the association between neighborhood measures of deprivation and primary hypertension diagnosis in youth.

**Design, Setting, and Participants:**

This cross-sectional study included 65 452 Delaware Medicaid-insured youths aged 8 to 18 years between January 1, 2014, and December 31, 2019. Residence was geocoded by national area deprivation index (ADI).

**Exposures:**

Higher area deprivation.

**Main Outcomes and Measures:**

The main outcome was primary hypertension diagnosis based on *International Classification of Diseases, Ninth Revision* and *Tenth Revision* codes. Data were analyzed between September 1, 2021, and December 31, 2022.

**Results:**

A total of 65 452 youths were included in the analysis, including 64 307 (98.3%) without a hypertension diagnosis (30 491 [47%] female and 33 813 [53%] male; mean [SD] age, 12.5 (3.1) years; 12 500 [19%] Hispanic, 25 473 [40%] non-Hispanic Black, 24 565 [38%] non-Hispanic White, and 1769 [3%] other race or ethnicity; 13 029 [20%] with obesity; and 31 548 [49%] with an ADI ≥50) and 1145 (1.7%) with a diagnosis of primary hypertension (mean [SD] age, 13.3 [2.8] years; 464 [41%] female and 681 [59%] male; 271 [24%] Hispanic, 460 [40%] non-Hispanic Black, 396 [35%] non-Hispanic White, and 18 [2%] of other race or ethnicity; 705 [62%] with obesity; and 614 [54%] with an ADI ≥50). The mean (SD) duration of full Medicaid benefit coverage was 61 (16) months for those with a diagnosis of primary hypertension and 46.0 (24.3) months for those without. By multivariable logistic regression, residence within communities with ADI greater than or equal to 50 was associated with 60% greater odds of a hypertension diagnosis (odds ratio [OR], 1.61; 95% CI 1.04-2.51). Older age (OR per year, 1.16; 95%, CI, 1.14-1.18), an obesity diagnosis (OR, 5.16; 95% CI, 4.54-5.85), and longer duration of full Medicaid benefit coverage (OR, 1.03; 95% CI, 1.03-1.04) were associated with greater odds of primary hypertension diagnosis, whereas female sex was associated with lower odds (OR, 0.68; 95%, 0.61-0.77). Model fit including a Medicaid-by-ADI interaction term was significant for the interaction and revealed slightly greater odds of hypertension diagnosis for youths with ADI less than 50 (OR, 1.03; 95% CI, 1.03-1.04) vs ADI ≥50 (OR, 1.02; 95% CI, 1.02-1.03). Race and ethnicity were not associated with primary hypertension diagnosis.

**Conclusions and Relevance:**

In this cross-sectional study, higher childhood neighborhood ADI, obesity, age, sex, and duration of Medicaid benefit coverage were associated with a primary hypertension diagnosis in youth. Screening algorithms and national guidelines may consider the importance of ADI when assessing for the presence and prevalence of primary hypertension in youth.

## Key Points

**Question:**

Is there an association between neighborhood measures of deprivation and hypertension diagnosis in youth?

**Findings:**

In this cross-sectional study of 65 452 Delaware Medicaid-insured youths aged 8 to 18 years between 2014 and 2019, residence in neighborhoods with a higher area deprivation index was associated with primary hypertension diagnosis.

**Meaning:**

These findings suggest that there is an association between greater neighborhood deprivation and a diagnosis of primary hypertension in youths, which may be an important factor to consider in assessing the presence and prevalence of hypertension in youths.

## Teaser

This cross-sectional study assesses the association between neighborhood measures of deprivation and diagnosis of primary hypertension in Medicaid-insured children and adolescents in Delaware.

## Introduction

It is estimated that hypertension is prevalent in 4 out of every 100 youth^1,2,3^ but underdiagnosed in 74% of cases.^3,4^ Hypertension can begin in childhood and is a predominant cause of target organ damage in childhood^5,6^ and cardiovascular disease (CVD) in adulthood.^7^ A diagnosis of hypertension is made in individuals younger than 13 years when the average blood pressure (BP) across 2 to 3 visits equals or exceeds the 95th percentile; in individuals 13 years of age or older, hypertension is diagnosed when the average BP equals or exceeds 130/80 mm Hg.^8^ Knowledge of factors associated with hypertension diagnosis in children and adolescents is essential to improving long-term cardiovascular outcomes.^7,9^

Previously published studies have highlighted a multitude of risk factors associated with primary hypertension development, including prenatal exposures,^10,11^ race,^12,13^ ethnicity,^12,13^ physical inactivity,^14^ increased sodium intake,^15^ lack of sufficient green space,^16^ environmental chemical exposures,^17^ ambient temperature,^17^ exposure to violence^18^ and crime,^19,20^ stress,^21^ low family income-to-poverty ratio,^12^ obesity,^22^ and lack of insurance.^23^ Studies outside the US have identified associations between parental education, occupation, employment, crowding, and home ownership and hypertension development in youth.^24^ However, to date, no US studies have evaluated the association between area deprivation index (ADI), an index of neighborhood-level socioeconomic factors, and primary hypertension diagnosis among insured US children and adolescents.

Physician recognition of hypertension is poor, even when BP values are consistent with the diagnosis.^3^ Current factors known to be associated with greater physician recognition of hypertension in youth include older age, male sex, higher BP, and obesity.^3^ However, the association between a child’s neighborhood-level deprivation and hypertension diagnosis has not been previously explored.

In this study, we assess the association between ADI and primary hypertension diagnosis in youth. We hypothesize that a higher ADI, even among Medicaid-insured youths, is associated with a greater likelihood of primary hypertension diagnosis.

## Methods

We performed a cross-sectional analysis of data from Delaware Medicaid recipients collected between 2014 and 2019. The Nemours Children’s Health and University of Delaware institutional review boards approved the study activities. A consent waiver was granted given that patients could not be identified directly or through identifiers. The study followed the Strengthening the Reporting of Observational Studies in Epidemiology (STROBE) reporting guideline.

### Cohort

We analyzed Delaware Medicaid administrative data, including medical claims, eligibility, and client data. The Delaware Division of Medicaid and Medical Assistance provides weekly data updates to the University of Delaware Center for Community Research and Service (CCRS). The CCRS accesses data for research and uses internal procedures to safeguard the data. For this study, CCRS policy scientists (M.J.M. and E.P.N.,) coded and provided aggregate data to the primary investigator (C.M.B.-S.).

### Study Population

Age of the patients included age at the end of the Medicaid enrollment year. We selected youths aged 8 to 18 years, similarly to a previously published study,^12^ due to reports of higher prevalence of hypertension among youths aged 8 to 9 years^2^ and to capture key developmental points for the diagnosis of hypertension from midchildhood through adolescence. We included youths with at least 1 health care visit (inpatient, outpatient, emergency department, long-term care, dental, or home health) and at least 1 month of full Medicaid insurance coverage between January 1, 2014, and December 31, 2019. We excluded pregnant youths, using approximately 388 pregnancy codes specified by the Office of Population Affairs.^25^

### Outcomes

The primary outcome was a diagnosis of primary hypertension by *International Classification of Diseases, Ninth Revision* (*ICD-9*) and *International Statistical Classification of Diseases and Related Health Problems, Tenth Revision* (*ICD-10*) codes, excluding a secondary hypertension diagnosis. We excluded secondary diagnoses because these conditions tend be less common^26^ and tend to present before age 6 years.^8^ Excluded from the primary hypertension outcome group were youths with *ICD-9* codes 402, 403, 404, and 405 and *ICD-10* codes I15 and I11.0. Included in the primary hypertension outcome group were youths with *ICD-9* codes 401.0, 401.1, and 401.9 and *ICD-10* codes I10 and I11.9. Choosing to prioritize sample size, no more than 1 encounter with a given *ICD-9* or *ICD-10* code was required for inclusion. We also did not specify the type of visit (eg, outpatient, inpatient, emergency department) or clinician type.

### Independent Variables

Each patient’s primary enrollment address, at the time of their first health care visit between 2014 and 2019, was geocoded using ArcGIS, version 10.8.1 (Esri). This census block group geocode was then used to link each participant with their 2014-2019 American Community Survey national ADI value by census block group.^27,28^

Updated every 5 years, the ADI is a composite index of 17 variables used to describe the socioeconomic status (SES) of the community in which one lives (Box). The ADI is based on weighted US census data and is not determined by race, ethnicity, or individual SES. It provides a geospatial description of neighborhood deprivation and ranking of area deprivation at the census block group level (eg, neighborhood) from 1 to 100, where an ADI of 100 represents communities with the most deprivation.

Box. **Seventeen Variables of the Area Deprivation Index**

Population aged ≥25 y with <9 y of education, %
Population aged ≥25 y with at least a high school diploma, %
Employed persons aged ≥16 y in white-collar occupations, %
Median family income, $
Income disparity
Median home value, $
Median gross rent, $
Median monthly mortgage, $
Owner-occupied housing units, % (home ownership rate)
Civilian labor force population aged >16 y unemployed, % (unemployment rate)
Families below poverty level, %
Population below 150% of the poverty threshold, %
Single-parent households with youth aged <18 y, %
Households without a motor vehicle, %
Households without a telephone, %
Occupied housing units without complete plumbing, %
Households with ≥1 person per room, % (crowding)

Data for the ADI are obtained from the American Community Survey.^28,29^ Delaware’s median national ADI is 38 (lower quartile, 25; upper quartile, 52).^30^ In this analysis, we included a dichotomous outcome variable of ADI greater than or equal to 50 (1) vs less than 50 (0), where ADI of 50 represents the upper quartile of the state’s national ADI and mean for the sample population.

Obesity diagnosis was ascertained using *ICD-9* codes 278.00 and 278.01 and *ICD-10* codes E66* (obesity). All youths with obesity had a Z code for body mass index (BMI, calculated by weight in kilograms divided by height in meters squared) greater than the 95th percentile or for BMI 30 and above (Z68.30-45). Any child with a Z code for BMI greater than the 95th percentile also had an *ICD-9* or *ICD-10* code for obesity.

Patient demographic characteristics (sex, race and ethnicity, and age) as described in the pooled Medicaid data set were included in the analysis. Delaware Medicaid data include self-reported race. For this study, race and ethnicity were categorized according to the following groups: Hispanic, non-Hispanic Black, non-Hispanic White, and other. Due to small individual subgroup sample size, youths with race and ethnicity described as Hawaiian; Indian or Alaskan; Native American; other, not Hispanic; or Pacific Islander were categorized as other.

We included a descriptor of the duration of Medicaid benefit coverage in months. An interaction term for ADI50 by months of Medicaid coverage was included to assess for an association between ADI50 and duration of Medicaid benefit coverage given that individual SES might influence the community in which one lives (ADI) and duration of Medicaid coverage. An additional interaction between ADI50 and obesity was assessed given publications highlighting an association between neighborhood deprivation and obesity.^16,31^

### Statistical Analysis

Data analysis was performed between September 1, 2021, and December 31, 2022. We provided descriptive statistics for demographic variables and used the χ^2^ test for comparison of categorical variables and *t* test for comparison of means of continuous variables (2-sided null hypothesis at an a priori significance level of *P* < .05). Based on univariable analyses, we selected age, biological sex, race and ethnicity (Hispanic, non-Hispanic Black, and non-Hispanic White), total months receiving Medicaid, national ADI50 (1 = at or above the state’s upper quartile of 50 vs 0 = below the state’s upper quartile of 50), and obesity diagnosis to model hypertension diagnosis. We ensured model fit using goodness-of-fit tests (deviance and Pearson correlation). All statistical analyses were performed using SAS, version 9.4 software (SAS Institute Inc). Receiver operating characteristic curve analysis was performed and area under the curve calculated for the final model.

## Results

### Study Population

**Figure 1.**
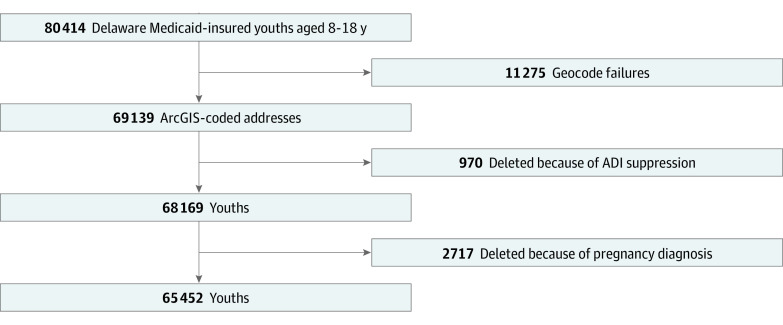
Delaware Medicaid-Insured Youths Aged 8 to 18 Years, 2014-2019 ADI indicates area of deprivation index; ADI suppression, geocoded addresses with no available ADI due to residence within high–group quarters population, low population, or low housing regions.

Of 80 414 Delaware Medicaid-insured youths aged 8 to 18 years, 69 139 (85.6%) had geocoded addresses. Geocode failures for 11 275 were secondary to post office box addresses, missing addresses, or absence of an available geocode. A total of 970 youths were removed because of ADI suppression due to high group quarters, low population, or low housing communities. Of the remaining youths, 2717 were removed because of a pregnancy diagnosis. A total of 65 452 youths were included in the final analysis (Figure 1). Mean (SD) duration of full Medicaid benefit coverage was 46.0 (24.3) months. Mean (SD) ADI was 50 (19). A total of 32 162 (49%) had an ADI greater than or equal to 50, and 33 290 (51%) had an ADI less than 50.

### Primary Hypertension Diagnosis

We identified 1145 (1.7%) youths with a diagnosis of primary hypertension (mean [SD] age, 13.3 [2.8] years; 464 [41%] female and 681 [59%] male; 271 [24%] Hispanic, 460 [40%] non-Hispanic Black, 396 [35%] non-Hispanic White, and 18 [2%] of other race or ethnicity) and 64 307 (98.3%) without a hypertension diagnosis (30 491 [47%] female and 33 813 [53%] male; mean [SD] age, 12.5 [3.1] years; 12 500 [19%] Hispanic, 25 473 [40%] non-Hispanic Black, 24 565 [38%] non-Hispanic White, and 1769 [3%] other race or ethnicity) (Table 1). Prevalence of primary hypertension was highest among youths aged 13 to 18 years. Among the youths with a primary hypertension diagnosis, 614 (54%) had an ADI greater than or equal to 50. The mean (SD) duration of Medicaid coverage was 60.6 (16.0) months for youths with a diagnosis of primary hypertension vs 46.0 (24.3) months for participants without a diagnosis of primary hypertension. Youth with a primary hypertension diagnosis were more likely to have an obesity diagnosis (705 [62%] vs 13 029 [20%]) (Table 1).

**Table 1.** Demographic Variables by Primary Hypertension Diagnosis^a^

	No. (%)		*P* value
	Hypertension diagnosis	No hypertension diagnosis	
No. of youths	1145 (1.7)	64 307 (98.3)	
Age, mean (SD), y	13.3 (2.8)	12.5 (3.1)	<.001
ADI			.002
≥50	614 (54)	31 548 (49)	
<50	531 (46)	32 759 (51)	
Duration of full Medicaid beneficiary coverage, mean (SD), mo	60.6 (16.0)	46.0 (24.3)	<.001
National ADI	52.7 (19.2)	50.2 (18.8)	
Obesity diagnosis			<.001
Yes	705 (62)	13 029 (20)	
No	440 (38)	51 278 (80)	
Race and ethnicity			<.001
Hispanic	271 (24)	12 500 (19)	
Non-Hispanic Black	460 (40)	25 473 (40)	
Non-Hispanic White	396 (35)	24 565 (38)	
Other^b^	18 (2)	1769 (3)	
Sex			<.001
Female	464 (41)	30 491 (47)	
Male	681 (59)	33 813 (53)	

Abbreviation: ADI, area deprivation index.

^a^ Categorical variables comparison by χ^2^ test and continuous variables comparison by *t* test. Significance set at a 2-sided *P* < .05. Three youths had missing data for sex.

^b^ Other includes Hawaiian; Indian or Alaskan; Native American; other, not Hispanic; and Pacific Islander.

#### Multivariable Logistic Regression

**Figure 2.**
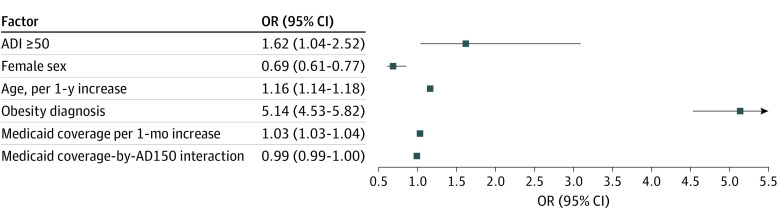
Final Multivariable Logistic Regression Model The sample size was 65 449 Medicaid-insured youths aged 8 to 18 years (<0.01% with missing data). Significance was set at a 2-sided *P* < .05. ADI indicates area deprivation index; ADI50, a dichotomous variable for national ADI greater than or equal to 50 vs less than 50.

According to multivariable analysis, patient-level factors associated with a greater likelihood of a primary hypertension diagnosis included ADI greater than or equal to 50 (odds ratio [OR], 1.61; 95% CI, 1.04-2.51; *P* = .03), older age (OR, 1.16; 95% CI, 1.14-1.18 for each additional year of age; *P* < .001), and obesity diagnosis (OR, 5.16; 95% CI, 4.54-5.85; *P* < .001). An interaction between ADI50 and obesity was not significant. Female sex was associated with a lower likelihood of a primary hypertension diagnosis (OR, 0.68; 95% CI, 0.61-0.77; *P* < .001) (Table 2 and Figure 2). Further analysis of the association between duration of Medicaid coverage and hypertension diagnosis when ADI was greater than or equal to 50 vs when ADI was less than 50 revealed ORs of 1.02 (95% CI, 1.02-1.03) and 1.03 (95% CI, 1.03-1.04), respectively (eTable in Supplement 1). The area under the curve for the final model was 0.79 (eFigure in Supplement 1).

**Table 2.** Analysis of Potential Factors Associated With Primary Hypertension Diagnosis^a^

	OR (95% CI)	*P* value
ADI ≥50	1.61 (1.04-2.51)	.03
Age, y	1.16 (1.14-1.18)	<.001
Hispanic	1.35 (0.83-2.20)	.22
Non-Hispanic Black	1.43 (0.89-2.31)	.14
Non-Hispanic White	1.44 (0.89-2.33)	.13
Female sex	0.68 (0.61-0.77)	<.001
Obesity diagnosis	5.16 (4.54-5.85)	<.001
Medicaid coverage, mo	1.03 (1.03-1.04)	<.001
Medicaid coverage-by-ADI50 interaction^b^	0.99 (0.99-1.00)	.04

Abbreviations: ADI50, area deprivation index greater than or equal to 50 or less than 50; OR, odds ratio.

^a^ Ten youths (0.02%) were missing from the analysis.

^b^ Interaction term between duration of Medicaid benefit coverage (mo) and ADI.

## Discussion

Cardiovascular disease remains the leading cause of death in the US.^32^ Hypertension is the leading modifiable risk factor for CVD development.^7^ In this study, we highlight a significant association between neighborhood deprivation and hypertension in youth.

Disparities in CVD prevalence plague the most vulnerable populations.^33^ We know and consider individual factors that influence risk for hypertension,^8,22,34^ yet we devise strategies for screening and diagnosis of hypertension that do not routinely consider neighborhood-level risk.^3,8,33,35,36^ In this cross-sectional study, we assessed the association between neighborhood measures of deprivation and diagnosis of primary hypertension in 65 452 Delaware youths insured by Medicaid. Consistent with our study hypothesis, we found that residence within a high-deprivation neighborhood was associated with 60% greater odds of a hypertension diagnosis. The association between ADI and primary hypertension diagnosis was rivaled only by a diagnosis of obesity, which was associated with 5 times greater odds of a primary hypertension diagnosis.

Our finding of an association between neighborhood and primary hypertension in youth is supported by previously published studies assessing the association between neighborhood deprivation and hypertension diagnosis in adults, environmental factors and hypertension diagnosis,^37,38,39^ childhood opportunity and cardiometabolic disease in midchildhood,^16^ and perinatal environmental exposures and BP in early childhood.^17^ According to the Residential Environment and Coronary Heart Disease cohort study of 5941 participants aged 30 to 79 years recruited in 2007-2008, systolic BP increased independently and regularly with both decreasing individual education and decreasing residential neighborhood education.^39^ In the Prospective Epidemiological Study of Myocardial Infarction, a European study surveying 7850 men aged 50 to 60 years, decreasing neighborhood education level was also associated with increased systolic BP.^38^ Early-life exposures to components of the built environment, including access to natural spaces, traffic, air pollution, noise, and meteorology, may be associated with hypertension prevalence at a young age. The Human Early Life Exposome project, a European consortium study, identified a positive association between levels of air pollution during the prenatal and postnatal periods and BP.^17^ High ambient temperature exposure and noise exposure during pregnancy were also associated with higher BP in offspring. There were limitations to these findings, including that the authors could not exclude a seasonal temperature effect and were unable to identify a noise dose-response trend.

The Child Opportunity Index combines 29 neighborhood-level indicators into a single composite measure to describe 3 domains of child health: education, health and environment, and social and economic. Aris et al^16^ geocoded the residential addresses of 743 youths and found that independent of individual and family SES, youths who resided in communities with higher overall opportunity (higher Child Opportunity Index, lower deprivation) had lower metabolic risk scores in midchildhood (7.9 years) and early adolescence (13.1 years).

The sensitive period model proposes that negative health exposures have a greater effect when they take place during sensitive developmental periods and influence later outcomes.^40^ Longitudinal studies of early-life exposure to low neighborhood SES and association with adult BP from the New England Family Study found that neighborhood deprivation may contribute to an increased risk for hypertension later in life.^41^ The study, however, did not assess the association between neighborhood SES and hypertension during childhood. Policies such as community redlining may also be adversely associated with BP and cardiovascular health status. Among adults, this process has been associated with higher rates of CVD and hypertension and poorer cardiometabolic health outcomes.^42^

Not all published studies, however, have identified an association between neighborhood deprivation and hypertension diagnosis in youth. Ribeiro et al^24^ assessed the relative index of inequality (RII), a summary measure of relative inequality that expresses the risk ratio of an outcome, comparing the outcome of the lowest social hierarchy group with the outcome of the highest social hierarchy group. In this study of 7459 youths aged 4 years living in Portugal, the RII for hypertension was greater than 1 with a 95% CI that did not include 1 when the SES indicator was parental (mother or father) education, occupation, income, or home ownership. The European Deprivation Index (EDI) was used in this study as another indicator of SES. The EDI is a cross-culture deprivation index used in 5 European countries. In Portugal, the EDI includes 8 variables. The EDI did not provide as detailed a description of the poverty level of the neighborhood households.^43^ Using this EDI, Ribeiro et al did not identify an RII for hypertension when the socioeconomic position evaluated was the EDI.

The ADI is specific to a smaller region and describes deprivation at the census block group level. The ADI provides a much more detailed description of the SES of the neighborhood than the EDI and at a more specific level (eg, census block group) than the Child Opportunity Index. In addition, the ADI is updated every 5 years.^27^

Insurance status is an important determinant of access to care.^23^ Without health insurance, many persons cannot receive regular medical care, and given that the diagnosis of hypertension relies on detection of a BP above an established threshold on 2 or more separate occasions,^8^ hypertension diagnoses can either be delayed or missed in the absence of insurance coverage. In this study of 65 452 Delaware Medicaid recipients aged 8 to 18 years, we assessed whether, beyond access to health insurance, which all the youths had, there was an association between duration of Medicaid insurance coverage and odds of a diagnosis of hypertension. We found greater odds of a primary hypertension diagnosis for each additional month of Medicaid insurance coverage that was slightly different in ADI less than 50 (OR, 1.033; 95% CI 1.027-1.038; *P* < .001) vs ADI greater than or equal to 50 (OR, 1.024; 95% CI, 1.019-1.029; *P* < .001) (eTable in Supplement 1). Without further data, we are unable to determine the exact reason for this difference.

Racial and ethnic disparities in hypertension prevalence have been well described, particularly in adults.^7,32^ Hypertension prevalence has been reported as highest among non-Hispanic Black men and women (56.5% and 55.3%, respectively) according to 2015-2018 National Health and Nutrition Examination Survey data.^7^ However, studies in children and adolescents have highlighted that racial and ethnic differences in hypertension prevalence are no longer significant when adjusted for BMI.^2^ Older studies, however, have suggested that even after adjusting for BMI, Hispanic males have higher odds of hypertension vs White males (OR, 1.21; 95% CI, 1.07-1.37; *P* < .01).^44^ We found no association between race and ethnicity and hypertension diagnosis.

### Limitations

This study has several limitations. First, we used an administrative database. Although the sample was large and diagnoses were available, BP and BMI values were not available. We know that 74% of cases of pediatric hypertension are underdiagnosed.^3,4^ Therefore, although our estimated prevalence of primary hypertension was similar to that of previously published reports,^2,3^ there may have been an underestimation of the true prevalence of hypertension in this population. Second, selection bias may have occurred, as we were forced to exclude 11 275 youths due to absence of geocoding. Nevertheless, our findings regarding the association between ADI and primary hypertension may remain valid given that we do not suspect a higher proportion of screen failures in more affluent communities and given that many of the youths with geocode failures may have resided within high-deprivation neighborhoods. Third, we excluded 2717 youths because of a pregnancy diagnosis. This exclusion also may not have contributed to selection bias as the exclusion was uniform and represented a low percentage of the total sample. Fourth, we assumed that youths had the same ADI throughout the 5-year study period. We had no reason to suspect that a significant number of youths changed addresses from a low- to a high- or a high- to a low-deprivation neighborhood during the 5-year study. Fifth, while we used very simplified categories for race and ethnicity, the distinctions used are those commonly reported in the literature. Future studies will more fully explore the association between distinct racial and ethnic groups and diagnosis of primary hypertension.

## Conclusions

The findings from this cross-sectional study highlight the significant association between neighborhood deprivation and a diagnosis of primary hypertension among Medicaid-insured youths and the importance of considering neighborhood-related factors, such as ADI, when diagnosing hypertension. Future studies are needed to further elucidate the association between the 17 components of ADI and hypertension development and diagnosis in youth. Screening algorithms and national guidelines may consider the importance of ADI when assessing for the presence and prevalence of primary hypertension in youth.

## Notes

Supplement 1. **eTable.** Assessment of Medicaid Duration and Area Deprivation Index (ADI) Interaction Term **eFigure.** Receiver Operating Characteristic Curve for Multivariable Logistic Regression Model

Supplement 2.
Data Sharing Statement
