# Supplementary material for: Association of Area Deprivation With Primary Hypertension Diagnosis Among Youth Medicaid Recipients in Delaware
Source: JAMA Netw Open. 2023 Mar 15;6(3):e233012. doi: 10.1001/jamanetworkopen.2023.3012 (PMC10018318; doi:10.1001/jamanetworkopen.2023.3012)
Supplement: Supplement 1. — eTable. Assessment of Medicaid Duration and Area Deprivation Index (ADI) Interaction Term eFigure. Receiver Operating Characteristic Curve for Multivariable Logistic Regression Model [file jamanetwopen-e233012-s001.pdf]

Supplemental Online Content

Baker-Smith CM, Yang W, McDuffie MJ, et al. Association of area deprivation with primary hypertension diagnosis among youth Medicaid recipients in Delaware. *JAMA Netw Open*. 2023;6(3):e233012. doi:10.1001/jamanetworkopen.2023.3012

eTable. Assessment of Medicaid Duration and Area Deprivation Index (ADI) Interaction Term

eFigure. Receiver Operating Characteristic Curve for Multivariable Logistic Regression Model

This supplemental material has been provided by the authors to give readers additional information about their work.

© 2023 Baker-Smith CM et al. *JAMA Network Open.*

eTable. Assessment of Medicaid Duration and Area Deprivation Index Interaction Term

	OR	95% Confidence Limit		*p-Value*
Age (years)	1.16	1.14	1.18	<0.001
Female Sex	0.69	0.61	0.77	<0.001
Medicaid Coverage (months), ADI 50=0	1.033	1.027	1.038	
Medicaid Coverage (months), ADI 50=1	1.024	1.019	1.029	
Obesity Diagnosis	5.14	4.53	5.82	<0.001

Legend: The odds of HTN diagnosis for each additional month of Medicaid full benefit coverage when the ADI is greater than of equal to 50. Medical coverage (months) ADI50=0, the odds of HTN diagnosis for each additional month of Medicaid benefit coverage when the ADI is less than 50.

© 2023 Baker-Smith CM et al. *JAMA Network Open.*

eFigure. Receiver Operating Characteristic Curve for Multivariable Logistic Regression Model

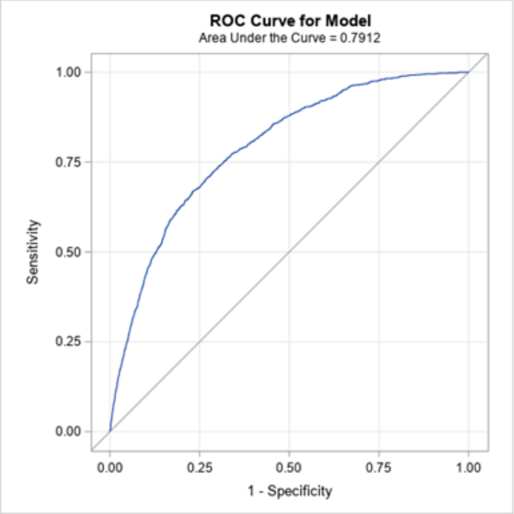

Legend: Outcome variable: Primary HTN diagnosis. Independent variables: age, sex, obesity diagnosis, ADI50, Medicaid duration, interaction term for Medicaid duration *ADI50

© 2023 Baker-Smith CM et al. *JAMA Network Open.*
