# Supplementary material for: Association of Area Deprivation With Primary Hypertension Diagnosis Among Youth Medicaid Recipients in Delaware
Source: JAMA Netw Open. 2023 Mar 15;6(3):e233012. doi: 10.1001/jamanetworkopen.2023.3012 (PMC10018318; doi:10.1001/jamanetworkopen.2023.3012)
Supplement: Supplement 2. — Data Sharing Statement [file jamanetwopen-e233012-s002.pdf]

Data Sharing Statement

Baker-Smith. Association of Area Deprivation With Primary Hypertension Diagnosis Among Youth Medicaid Recipients in Delaware. *JAMA Netw Open*. Published March 15, 2023. doi:10.1001/jamanetworkopen.2023.3012

Data
Data available: Yes
Data types: Other (please specify)
Additional Information: Due to rules and restrictions of Delaware State Medicaid, data from this study cannot be shared.
How to access data: Due to rules and restrictions of Delaware State Medicaid, data from this study cannot be shared.
When available: With publication

Supporting Documents
Document types: None

Additional Information
Who can access the data: Due to rules and restrictions of Delaware State Medicaid, data from this study cannot be shared.
Types of analyses: Due to rules and restrictions of Delaware State Medicaid, data from this study cannot be shared.
Mechanisms of data availability: Due to rules and restrictions of Delaware State Medicaid, data from this study cannot be shared.
Any additional restrictions: Due to rules and restrictions of Delaware State Medicaid, data from this study cannot be shared.
